# Chemical Profiling and Biological Activity of Extracts from Nine Norwegian Medicinal and Aromatic Plants

**DOI:** 10.3390/molecules27217335

**Published:** 2022-10-28

**Authors:** Rune Slimestad, Amritha Johny, Mette Goul Thomsen, Christian Renè Karlsen, Jan Thomas Rosnes

**Affiliations:** 1 PlantChem AS, Eikenveien 334, N-4596 Eiken, Norway; 2 Department of Fish Health, Nofima AS, Osloveien 1, N-1430 Ås, Norway; 3 Division of Food Production and Society, Nibio, Nylinna 226, N-2849 Kapp, Norway; 4 Department of Processing Technology, Nofima AS, Richard Johnsens gate 4, N-4021 Stavanger, Norway

**Keywords:** herbs, solvent extraction, TEAC, total phenolics, UHPLC-MS, antimicrobial

## Abstract

There is an increased interest in identifying beneficial compounds of plant origin that can be added to animal diets to improve animal performance and have a health-promoting effect. In the present study, nine herb species of the Norwegian wild flora or which can be cultivated in Norway were selected for phytogenic evaluation (hops, maral root, mint, oregano, purslane, rosemary, roseroot, sweet wormwood, yarrow). Dried herbs were sequentially extracted with dichloromethane (DCM), ethanol (EtOH) and finally water (H_2_O) by ultrasound-assisted extraction (UAE). The UAE protocol was found to be more rational than conventional Soxhlet with respect to DCM extraction. Total extraction yield was found to be highest for oregano (*Origanum vulgare*) with 34.4 g 100^−1^ g dry matter (DM). H_2_O-extracts gave the highest yields of the three solvents, with up to 25 g 100^−1^ g DM for purslane (*Portulaca oleracea* ssp. sativa) and mint (*Mentha piperita*). EtOH- and H_2_O-extracts were the most efficient extracts with respect to free radical scavenging capacity (ABTS (=2,2-azino-bis (3-ethylbenzothiazoline-6-sulfonic acid), and oregano, mint, hops (*Humulus lupulus*) and maral root-leaves (*Leuzea carthamoides*) were found to be the most efficient antioxidant sources. Hops (EtOH-extract) contained α- and β-acids, xanthohumols, chlorogenic acid and the hitherto unreported 3-*O*-glucosides of kaempferol and quercetin. Maral root-leaves contained among other compounds hexosides of the 6-hydroxy- and 6-methoxy-kaempferol and -quercetin, whereas roseroot (*Rosea rhodiola*) revealed contents of rosavin, rhodiosin and rhodionin. Sweet wormwood (*Artemisia annua*) contained chlorogenic acid and several derivatives thereof, scopoletin and poly-methylated flavones (eupatin, casticin, chrysoplenetin). Antimicrobial potential of different plant extracts was demonstrated against Gram-positive and Gram-negative bacteria using the indicator organisms *Staphylococcus aureus*, and *Escherichia coli*, and the Atlantic salmon bacterial pathogens *Moritella viscosa*, *Tenacibaculum finnmarkense* and *Aliivibrio wodanis*. DCM extracts possessed the highest activities. Data demonstrate the potential ability of herb extracts as natural antimicrobials. However, future safety studies should be performed to elucidate any compromising effect on fish health.

## 1. Introduction

There exist several definitions of the term ‘herb’ which is derived from the Latin ‘herba’, meaning ‘green crop’ or herbage [1]. Botanists use ‘herb’ to describe non-woody plants, whereas a horticulturist uses the most familiar definition: a plant that may be used for fragrance, flavour, medicine, or dye [1]. Several herbs are recognized due to their high content of secondary metabolites like essential oils, phenolics, terpenes and alkaloids. External factors like light and temperature have shown to stimulate production of bioactive compounds in herbs under a controlled environment [2,3]. Due to Norway’s latitude with more daylight hours during the production season and high differences in day-night temperatures, herbs are stimulated to produce high amounts of secondary metabolites [4]. 

Phytogenic feed additives have gained interest due to antioxidant and antimicrobial properties, and some compounds have been found with healing properties against a wide range of fish pathogens including bacteria and parasites [5,6,7,8]. Several plant species are recognised for these activities like sweet wormwood (*Artemisia annua*), peppermint (*Mentha piperita*), thyme (*Thymus vulgaris*), marjoram (*Origanum majorana*), oregano (*Origanum vulgare*), yarrow (*Achillea millefolium*), and hops (*Humulus lupulus*) [9,10]. Moreover, there is considerable concern about the risk of ulcerative diseases caused by Atlantic salmon skin ulcer bacteria in on-growing salmon in Norway. Classical winter ulcer is typically related to the infection by *M. viscosa*. *Tenacibaculosis* by *Tenacibaculum* spp. is classified as non-typical winter ulcer. However, there is a third bacterial species, *A. wodanis* which itself is not ulcer causing pathogen but is always co-isolated with *M. viscosa* [11]. These pathogens alone or together can increase the spread of disease in salmon during the sea phase and remains as a major cause for mortality, reduced production quality and reduced fish welfare [12]. Thus, new eco-friendly methodologies that could limit or inhibit the growth of aquaculture pathogenic bacteria are of high interest. However, little is still understood about which crude herb extracts that exhibit promising antimicrobial activity against seawater pathogens of Atlantic salmon. Oxidation of lipids and proteins is the major mechanism of impaired quality of stored feed, and rosemary, oregano, and thyme are examples of herbs used in this context [13]. Plant-based antioxidants are mainly phenolic compounds, carotenoids, and vitamins [8], and due to their ubiquity and often high concentrations, herb phenolics are recognized as important antioxidants. 

The varying effectiveness of vaccines, use of antibiotics and increasing antimicrobial resistance in case of disease outbreaks, along with the ban on synthetic antioxidants, has led to search for non-toxic natural alternatives to replace synthetic compounds in salmonid farming. Plant-based bioactive extract and compounds can be a good replacement due to availability and low production cost when compared to other commercially available alternatives. It is thus necessary and critical to develop suitable methods to extract bioactive compounds from herbs, followed by high-throughput screening of metabolites to identify potential compounds present in the plants. 

During phytogenic extraction, the method of choice will have a massive impact on the yield and quality of the final extract [14]. The method should, for rational causes, give high yields and in addition, should be highly selective toward a wide range of bioactive compounds, dissolving the molecules of interest and leaving the rest as undissolved material. A variety of solvents with different polarity and other characteristics are used for extraction of non-volatile compounds, and some of these are used in combination with physical methods (e.g., electrical field, microwave, ultrasound, pressure, heat) or arranged in specific manners (e.g., accelerated solvent, Soxhlet) to improve the effectiveness of the extraction process [15]. Ultrasound Assisted Extraction (UAE, sonication) induces phenomena in the medium, such as acoustic streaming and cavitation, which leads to intense agitation and mass transfer enhancement [16]. UAE techniques accelerate the release of essential oils from aromatic plants as they facilitate the penetration of the solvents in the plant material [17].

Despite the traditional use of herbs as medical and aromatic plants, only a limited number of species are regularly grown in Norway, and a scarce number have been applied as feed ingredients to enhance shelf-life and to add an antibacterial benefit to the feed. To the best of our knowledge, this is the first comprehensive work on screening of Norwegian grown herbs as potential phytogenic feed additives. This is a preliminary study conducted as a part of an ongoing survey to evaluate the potential of Norwegian grown herbs for phytogenic feed utilization (https://nofima.com/projects/bioactive-herbs-for-feed-and-packaging/ accessed 25 October 2022). Here, we have screened DCM, EtOH and H_2_O extracts from nine herbs grown in Norway for their chemical content and their antioxidant properties. The extracts were also analyzed for their antimicrobial effects based on their inhibition against the Atlantic salmon skin pathogens, *A. wodanis*, *M. viscosa*, and *T. finnmarkense*.

## 2. Results and Discussion

**Figure 1:**
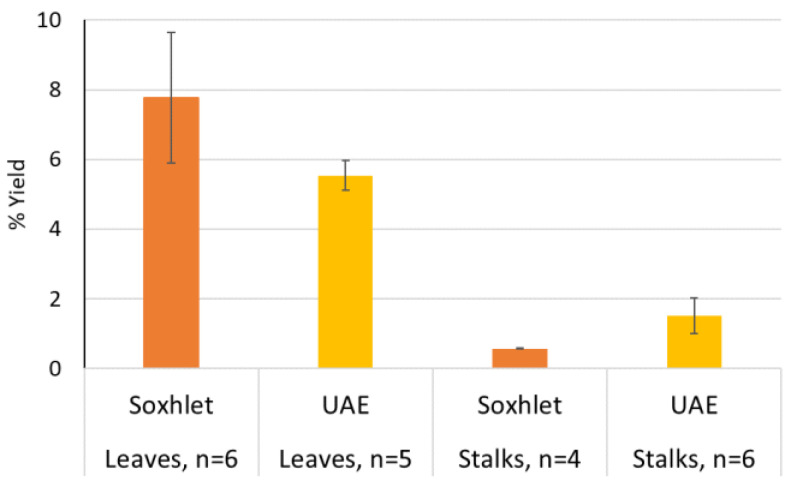
Percentage yield (mean ± SD) of DCM-extracts of leaves and stalks of sweet wormwood.

Comparison of DCM extraction methods. Yields from Soxhlet extraction were compared to those obtained by UAE of leaves and stalks of sweet wormwood (Figure 1). The number of cycles or extractions determined by visual decolourisation of the extracts (chlorophylls), showed a total extraction time of 70 min per sample for both techniques. Soxhlet was found to give significantly higher yields of leaf-extracts, compared to the UAE method (95% CI, 9 DF, *p* = 0.0293). For stalks an even more pronounced but opposite effect was found with the use of UAE compared to Soxhlet (95% CI, 8 DF, *p* = 0.0065). Altogether, UAE was chosen as the extraction method for DCM extracts throughout this work as it also is compatible with EtOH and H_2_O extraction, whereas the latter solvent is not in agreement with the use of Soxhlet as an extraction method [18]. 

UAE with methanol has previously been used to optimize the extraction of five sesquiterpenes from sweet wormwood leaves [19]. That method included two extraction cycles but at only 15 min each, but with a higher material to liquid ratio (1:20 g mL^−1^). Extraction temperature was in the present case in the range 30–40 °C. Higher temperatures generally lead to a higher solvent solubility capacity, a lower viscosity improving solvent penetration into plant cells, and to a reduction in solute-matrix interactions. All these effects lead to an improvement in the extraction yield but can also lead to a decrease in selectivity [15]. Soxhlet has traditionally been widely applied as an extraction method with extraction solvents of low boiling points, and has recently been used for extraction of essential oils as well as phenolics from rosemary and chamomile [20,21]. The method was found to be more efficient with respect to total extraction yield and total extracted phenolics compared to the use of maceration with methanol. To the best of our knowledge, the present report contains however the first direct comparison between Soxhlet and UAE with respect to DCM extraction yield, and demonstrates that UAE is superior to Soxhlet in the case of stalks of sweet wormwood but not in case of leaf extraction.

**Figure 2:**
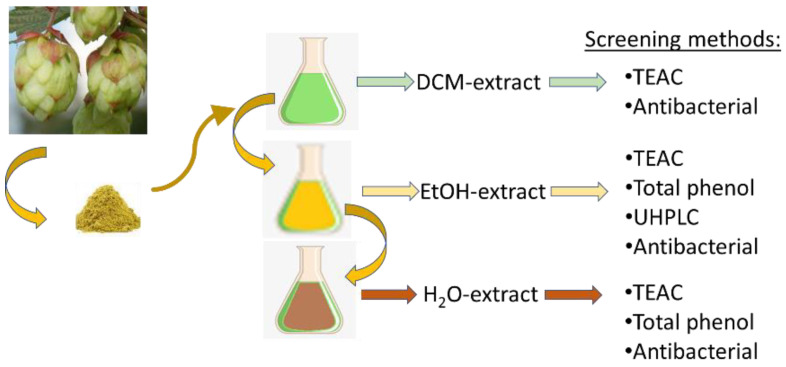
Diagram of sample treatment, sequential extraction, and screening methods for each extract.

The initial screening of antioxidant and antimicrobial activities of herbs in this work was performed in a non-targeted manner. The method included successive extractions with increasing polarity of the extraction solvents. Thus, extraction with DCM was followed by EtOH extraction and H_2_O (Figure 2). 

**Figure 3:**
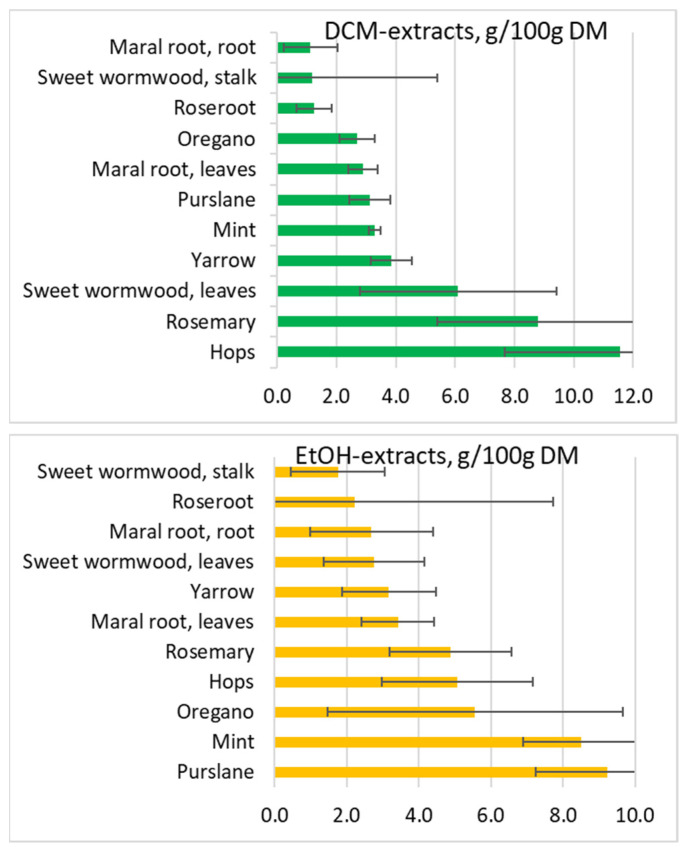

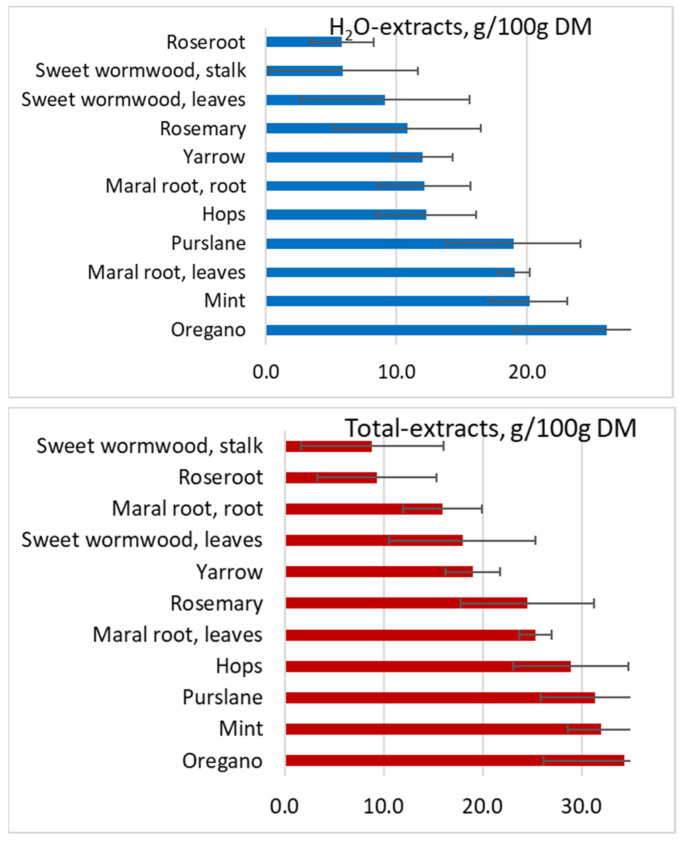
Eleven herbal extracts arranged by increasing yields (g 100 g^−1^ DM ± SD) for three sequential extraction steps: DCM (**green**) followed by EtOH (**yellow**) and H_2_O (**blue**). Total extraction yield of all three samples (DCM + EtOH + H_2_O) (**red**).

Extraction yields. Higher yields were obtained with polar solvents in particular with H_2_O. However, hops and rosemary gave high yields with DCM extraction, whereas mint and purslane exhibited high yields with EtOH (Table 1, Figure 3). Both hops, maral root leaves, mint and purslane obtained total extraction yields above 25% (mass-to-mass ratio). 

Radical scavenging activities. The ABTS free radical-scavenging activities were, in general, found to be highest in the H_2_O- and lowest in the DCM-extracts (Table 2). Among the nine species, oregano followed by mint, roseroot and hops gave the highest TEAC-values of the H_2_O-extracts. Among the EtOH-extracts hops followed by roseroot, maral root leaves, and sweet wormwood leaves (from the September harvest), revealed highest activities, whereas rosemary and hops were the strongest ABTS-scavengers among the DCM-extracts. 

Calculation of radical scavenging capacities based on plant DM (values from Table 2 adjusted for yield) revealed the most optimal plant species and extraction solvent with respect to antioxidant activities (Table 3). H_2_O was in general found to be the most efficient extraction solvent of free radical scavengers. Oregano followed by mint, hops, rosemary and maral root leaves were found to be the most optimal species. 

The phenolic content among the EtOH-extracts was highest in hops and roseroot (Table 2), whereas oregano demonstrated exceptional amounts of phenolics in the H_2_O-extracts in particular on a dry plant matter basis (Table 3). Phenolic content in sweet wormwood increased from June to September in both leaves and stalks. This result points out the importance of a late harvest of these plants with respect to phenolic content. The ABTS-scavenging capacities for all extracts correlated with total phenolic content both for the EtOH-extracts (corr = 0.88) and the H_2_O-extracts (corr = 0.98).

### UHPLC-Screening of EtOH-Extracts

**Figure 4:**
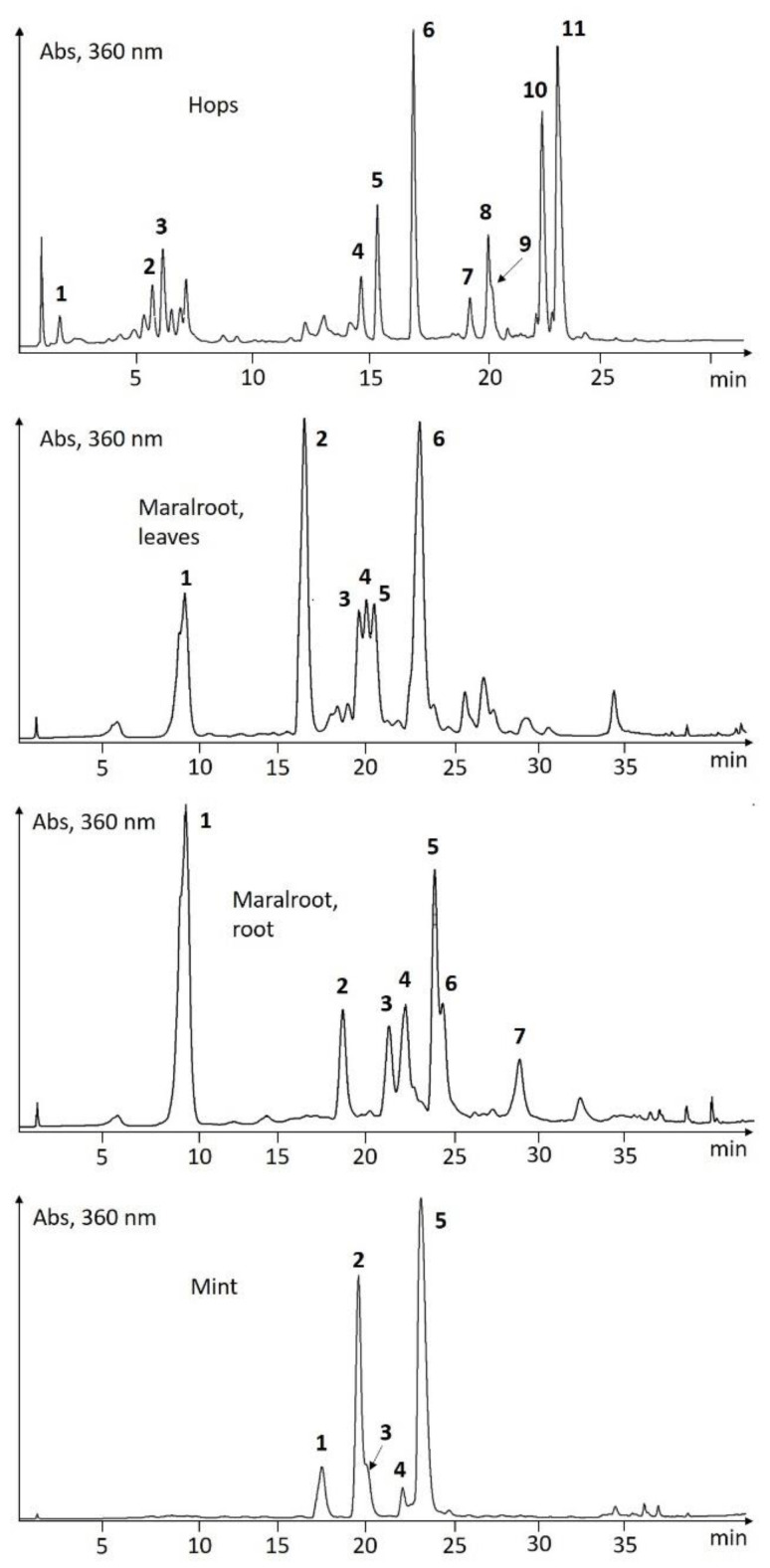

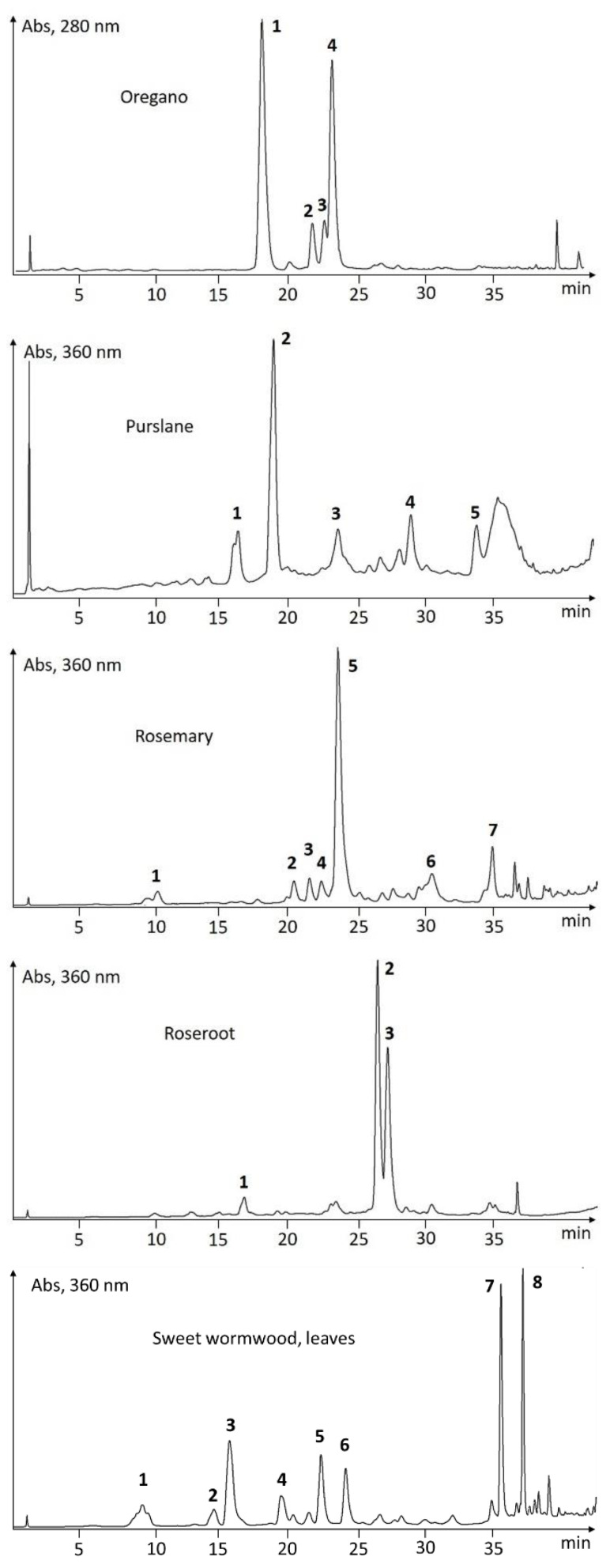

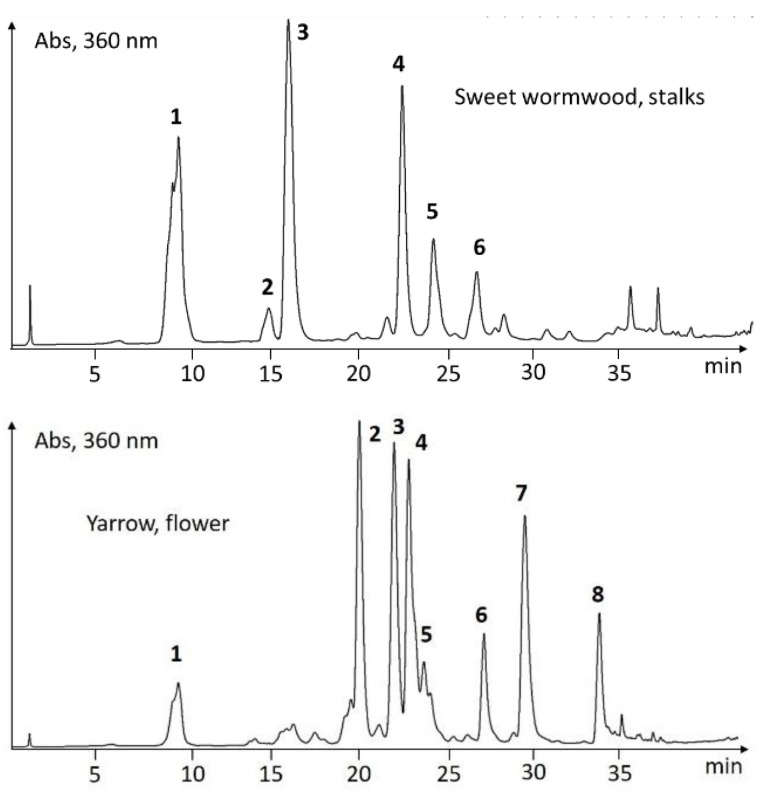
Chromatograms obtained by UHPLC-DAD of EtOH-extracts from nine herb species. Peaks are characterised according to Table 4, and peak numbers are restarted in each chromatogram.

**Figure 5:**
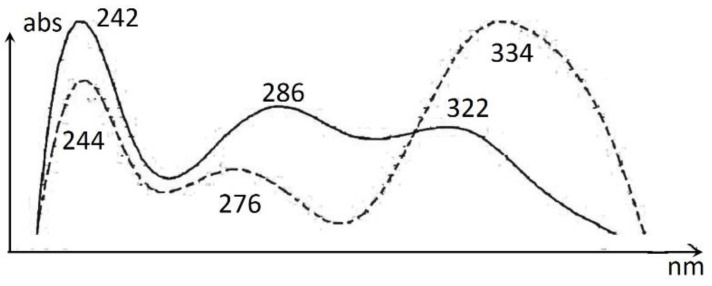
Spectra revealing the difference in UV-absorbance of α- (continuous) and β- (dotted) bitter acids in hops.

Hops: Xanthohumol C and bitter acids (α- and β-acids) were found to be the major compounds in addition to minor amounts of xanthohumol B, desmethylxanthohumol C, chlorogenic acid and flavonols (Figure 4 Table 4). Chlorogenic acid, kaempferol- and quercetin-3-glucoside were confirmed by co-chromatography with authentic samples. The phenolic content of female hop cones has been described previously [22,23], but few reports have described it from Norwegian growth practices. The content of flavonols is scarcely reported. Humulone and adhumulone have similar spectral features, and their peak assignments were done on the assumption that humulone is the major α-acid, and that it has lower retention on the C18-column [24]. The assignment of the α- versus the β-series is based on the mass difference of 52 amu (atomic mass units) for the respective three couples of compounds. There is also a noticeable difference in the UV-spectra of α- versus β-acids which can be used for assigning the series (Figure 5).

Maral root, leaves: Six major peaks appeared in the chromatogram of the EtOH-extract (Figure 4). They were putatively assigned as chlorogenic acid in addition to five flavonol structures based on chromatographic appearance and UV-spectra (Table 4). The absorbance of peaks 2, 3, 5 and 6 revealed an oxygenation pattern of ring A (of a flavonol) which is consistent with three O-substituents at the 5-position, the 6- or 8-position and at the 7-position due to an additional absorbance peak within Band II of the absorbance spectrum [25]. As oxygenation of position 8 typically gives a bathochromic shift of Band I compared to the regular 5,7-OH substitution pattern, whereas oxygenation of position 6 does not, ring A of peaks 2, 3, 5 and 6 was assigned with oxygenation at positions 5, 6 and 7 [26]. Peak **2** revealed pseudomolecular ions at *m*/*z* 481 and 479 in the positive and negative ionization mode, respectively. A fragment ion at *m*/*z* 319 correspond to [M-162+H]^+^ and one at *m*/*z* 163 to that of [M-318+H]^+^, which is in agreement with 6-hydroxyquercetin-*O*-hexoside. The structure of compound **3** was revealed in an analogous manner, but with a mass difference of -16 amu to that of peak **2**, proposing the identity to be 6-hydroxykaempferol-*O*-hexoside. The base peak in the mass spectrum of compound **5** was *m*/*z* 333, which corresponds to [M-162+H]^+^ indicating an additional methyl-ether group of the aglycone moiety compared to that of compound **2**. Compound **5** was assigned to be 6-methoxyquercetin-*O*-hexoside in accordance with the report on 6-methoxyquercetin (=patuletin) from this species [27]. In analogy, compound **6** appeared with an aglycone with a mass difference of -16 amu to compound **5**, which characterize compound **6** as 6-methoxykaempferol-*O*-hexoside. 

Maral root: The UV-spectrum of peak **2** revealed the compound to be a flavonol with an oxygenation pattern at positions 3′,4′ (and eventually 5′). The pseudomolecular ion at *m*/*z* 579, together with the fragment-loss of 162 amu ([M-162+H]^+^) is in accordance with isorhamnetin *O*-hexoside. The other assigned peaks revealed UV spectra in accordance with that of caffeic acid (Table 4). Based on mass spectral data, compound **1** was assigned to be chlorogenic acid, whereas compounds **3**–**5** were tentatively identified as isomers of di-caffeoylquinic acid. None of the five flavonols reported as part of this screening has to the best of our knowledge been reported from maral root previously. However, Stodulka and co-workers reported on the occurrence of patuletin (=6-methoxyquercetin) and 6-hydroxykaempferol-7-*O*-(6″-*O*-acetyl-β-D-glucopyranoside) from leaves [27]. However, there is no published evidence based on NMR or co-chromatography with authentic standards for the glycosylation positions nor for the nature of the glycoside. 

Mint: Five peaks were detected in the 360 nm window of the EtOH-extract. The UV spectrum of peak **1** was typical for those of dihydroflavonols and flavanones with a major absorbance band at 282 nm [26]. Its pseudomolecular ions at *m*/*z* 597 and 595 in the positive and negative ionisation modes, respectively, and its loss of two glycoside units (-146 and -162 amu, respectively) together with an aglycone at *m*/*z* 289 is in agreement with eriodictyol 7-*O*-rutinoside (=eriocitrin) [28]. Compound **2** contained an extended chromophore compared to compound **1**, and the full structure is in agreement with luteolin 7-*O*-rutinoside [28]. Peak **3** had a nearly identical UV spectrum to that of compound **2** and with a similar mass of the aglycone, 286 amu. The glycoside-fragment had a mass of 176 amu, which is consistent with that of a glucuronic acid, thus peak 3 was assigned to be luteolin *O*-glucuronide. Rutin (4) and rosmarinic acid (5) were assigned according to their retention times and typical mass spectral characteristics. Mint is recognized to be a rich source of rosmarinic acid, in addition to several structures of flavones and flavanones [28]. There are also reported a minor number of flavonols, included rutin (=quercetin 3-*O*-rutinoside).

**Figure 6:**
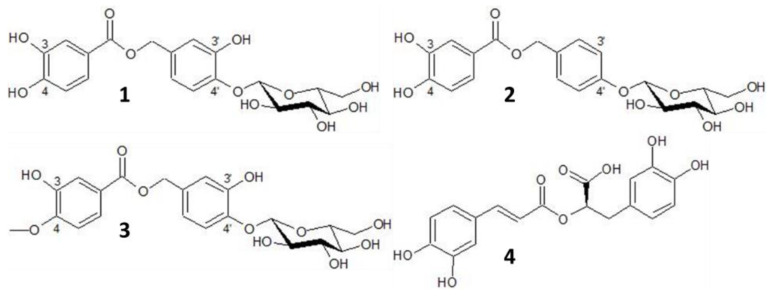
Chemical structures of the four main phenolic compounds in the EtOH-extracts of oregano: 4′-*O*-β-D-glucopyranosyl-3′,4′-dihydroxybenzyl protocatechuate (oreganol A) (**1**), 4′-*O*-β-D-glucopyranosyl-4′-hydroxybenzyl protocatechuate (**2**), 4′-*O*-β-D-glucopyranosyl-3′,4′-dihydroxybenzyl 4-*O*-methylprotocatechuate (oregano B) (**3**), rosmarinic acid (**4**).

Oregano: The UHPLC chromatogram was obtained at the 280 nm detection window and revealed four major peaks. The pseudomolecular ion in negative mode of peak **1** revealed *m*/*z* 437 ([M-H]^−^) and 875 ([2M-H]^−^). Loss of glucose was found as the fragment ion at *m*/*z* 275 ([M-glc-H]^−^), whereas the ion at *m*/*z* 153 is in agreement with dihydroxybenzoate. The spectrum agrees with that of oreganol-A (4′-*O*-β-D-glucopyranosyl-3′,4′-dihydroxybenzyl protocatechuate) as first isolated by Matsuura and co-workers [29]. For peak **2**, the MS- spectrum revealed pseudomolecular ion at *m*/*z* 421 ([M-H]^−^) and 843 ([2M-H]^−^). Loss of glucose was found as the ion fragment *m*/*z* 275, whereas the ion at *m*/*z* 153 is in accordance with dihydroxybenzoate 4′-*O*-β-D-glucopyranosyl-4′-hydroxybenzyl protocatechuate [30]. Compound **3** appeared with a pseudomolecular ion at *m*/*z* 451 ([M-H]^−^) and 903 ([2M-H]^−^). The ion fragment at *m*/*z* 275 was assigned to be [M-glc-H]^−^, whereas the ion at *m*/*z* 167 was in accordance with methoxy-hydroxybenzoate. The spectral characteristics are in line with the structure of oreganol-B (4′-*O*-β-D-glucopyranosyl-3′,4′-dihydroxybenzyl 4-*O*-methylprotocatechuate), which was isolated by Matsuura and co-workers [29]. The main ion fragment of compound **4** in the ESI+ mass spectrum was *m*/*z* 163, which is in accordance with a caffeoyl unit. In the ESI- spectrum [M-H]^−^ and [2M-H]^−^ were detected at *m*/*z* 359 and 71, respectively. The structure was thus assigned to be rosmarinic acid (Figure 6).

**Figure 7:**
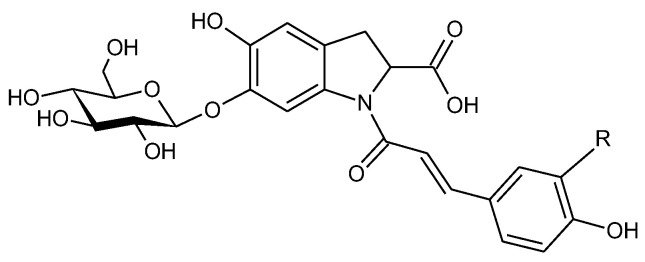
Molecular structures of oleracein W (R=OH) obtained as peak **1** and oleracein A (R=H) as peak **2** of the chromatogram of purslane.

Purslane: The UHPLC-chromatogram of the EtOH-extract displayed five major compounds detected at 360 nm (Figure 4). Peaks **1**, **2** and **4** showed an odd number of nitrogen atoms as part of their molecular structures due the even-numbered pseudomolecular ions (Table 4). Peak **1** revealed pseudomolecular ions at *m*/*z* 520 and 518 at ESI+ and ESI-, respectively, and was putatively assigned as oleracein W-hexoside [31]. The mass spectrum of peak **2** gave pseudomolecular ions at *m*/*z* 504 and 502 (ESI+ and ESI-) and a characteristic fragment ion at *m*/*z* 342 [M-hex+H]^+^. The MS features and the UV spectrum were in accordance with that of oleracein A (Figure 7) [32]. Compounds **4** and **5** exhibited similar UV spectra to that of compound **2**. In accordance with the nitrogen rule, compound **4** contained an odd number of nitrogen atoms, and was thus assigned to be an oleracein-derivative. On the contrary, compound **5** did not reveal odd numbers of nitrogen, and was assigned to be a caffeic acid derivative. 

Alkaloids have been reported to be important chemical constituents of purslane. In particular, it contains N-*trans*-feruloyltyramine [33], dopamine, dopa and a high concentration of noradrenaline [34]. In addition to oleracein W-*O*-hexoside and oleracein A, a third peak appeared with similar UV spectrum and an odd number of N-atoms. In accordance with its pseudomolecular mass at *m*/*z* 708, the compound might be *N*-sinapoyl 5,6-dihydroxyindoline-2-carboxylic acid acylated with coumaric acid and with a glucose (sin-glc-ind-coum; SGIC), or *N*-feruoyl 5,6-dihydroxyindoline-2-carboxylic acid acylated with another ferulic acid and appearing as a glucoside (fer-glc-ind-fer; FGIF) [31]. SGIC would probably exhibit absorbance maximum at higher wavelength than what was detected, and the peak was thus tentatively assigned to be FGIF. The 5,6- dihydroxyindoline-2-carboxylic acid, or the indoline core, characteristic for every oleracein, is represented with fragment ions 194.0, 150.1, and 148.0 *m*/*z*, in decreasing intensity. Fragment 194.0 *m*/*z*, as well as the characteristic fragment ions for the hydroxycinnamic acids are more prominent in oleraceins with lower mass [31].

Rosemary: The major phenolic compound in the EtOH-extract of rosemary was rosmarinic acid (peak 5, Figure 4). Minor compounds were flavonoids and caffeic acid derivatives (Table 4). The UV spectrum of peak **3** was in accordance with that of a flavone but with two absorbance peaks in Band II (250–290 nm) with similar intensities, which indicate oxygen substituents in positions 5, 6 and 7 of the A-ring. The pseudomolecular ion at *m*/*z* 479 was confirmed by its sodium adduct ion at *m*/*z* 501. The fragment ions at *m*/*z* 317 and 163 were in agreement with the loss of a hexose-unit; [M-162+H]^+^ and [M-317+H]^+^, respectively. The aglycone with *m*/*z* 317 is in agreement with luteolin substituted with a methoxy group. The structure was putatively assigned to be 6-methoxyluteolin 7-glucoside (=nepitrin). Peak **4** revealed a pseudomolecular ion, [M-H]^−^, at 515 (base peak), and a fragment ion at *m*/*z* 353, indicating the loss of a caffeoyl moiety to give chlorogenic acid. In positive mode, the spectrum revealed the fragments [M-H_2_O+H]^+^ at *m*/*z* 499, [M-162+H]^+^ at *m*/*z* 355 and [M-355+H]^+^ at *m*/*z* 163. The compound was thus assigned to be a dicaffeoylquinic acid. Peak **6** revealed an UV spectrum similar to that of peak **3**, and the base peaks in both MS ionization modes were in accordance with a methoxy-tetrahydroxy substitution pattern of the aglycone; *m*/*z* 317 and 315, respectively, thus the compound was assigned as nepetin (=6-methoxyluteolin). Rosemary is known to contain carnosolic acid, carnosol, carnosic acid, epirosmanol, rosmanol, methylcarnosate and isorosmanol in addition to essential oils [35]. These compounds were not detected as major compounds in the EtOH-extracts, and might have been removed through the first DCM-extraction. Upon reversed phase chromatography (C18-column) the phenolic diterpenes and triterpenes have higher retention than rosmarinic acid [36], and none of the appearing peaks had spectral characteristics in accordance with these terpenes. Peaks in the chromatogram of rosemary (Figure 4) were characterised as shown in Table 4 and assigned in accordance with literature [37,38]. The occurrence of nepitrin (=6-methoxyluteolin-7-*O*-glucoside, peak 3) is rather infrequent, and this is to the best of our knowledge the first report on both nepitrin and nepetin from rosemary. Nepitrin has been isolated from *Salvia plebeia* [39].

Roseroot: One minor and two major peaks appeared in the chromatogram of the EtOH-extract (Figure 4). Peak **1** revealed an adduct ion at *m*/*z* 446 [M+NH_4_]^+^ and a pseudomolecular ion at *m*/*z* 427 [M-H]^−^ and was assigned to be rosavin [40]. The major compounds (peaks **2** and **3**) contained an aglycone with UV absorption spectra typically for flavonols with only one hydroxyl substituent on the B-ring, but with an additional absorption band at 330 nm. The fragments at *m*/*z* 303 and 301 were assigned to belong to that of the aglycone ([agl+H]^+^ and [agl-H]^−^, respectively). The pseudomolecular ion at *m*/*z* 611 and 609, together with the fragment ion at 449, is in accordance with rhodiosin (=8-OH-kaempferol 7-*O*-glucorhamnoside), whereas the pseudomolecular ion at *m*/*z* 449 and 447 is in line with that of rhodionin (=8-OH-kaempferol 7-*O*-rhamnoside or herbacetin 7-*O*-rhamnoside) [40]. 

Sweet wormwood, leaves: The compounds detected from the EtOH extract of leaves were putatively identified according to Table 4. Peaks 1, 2, 5 and 6 revealed UV absorbance spectra in accordance with that of caffeic or ferulic acids, and compound 1 was assigned to be chlorogenic acid based on co-chromatography with an authentic compound. Peak **2** revealed the pseudomolecular ion at *m*/*z* 367 [M-H]^−^ which was confirmed by the presence of the base peak at *m*/*z* 735, which is in accordance with [2M-H]^−^. The fragment ion at *m*/*z* 177 is typical for ferulic acid, and peak **2** was thus assigned to be feruloylquinic acid. Peaks **5** and **6** exhibited similar spectral features, and based on the pseudomolecular ions, loss of water (*m*/*z* 499; [M-18+H]^+^) and the fragments typical for caffeic acid (163 amu), the compounds were assigned to be isomeric structures of dicaffeoyl quinic acid. Peak **5** and **6** exhibited closely related chromatographic and spectral features, which were in accordance with that of dicaffeoylquinic acid, and the two compounds were thus assigned to be isomeric compounds thereof. Peak **3** gave an UV absorbance spectrum similar to that of a 6,7-di-oxy-coumarin [41]. The base peak of the ESI+ spectrum was found to be the pseudomolecular ion at *m*/*z* 193 confirmed by the presence of [M+Na+H]^+^ at *m*/*z* 215 and [M-H]^−^ at *m*/*z* 191. The peak was characterised to be scopoletin. Peak **7** and **8** appeared late in the chromatogram indicating a hydrophobic molecular character. Both compounds appeared to be flavonols based on the UV absorption spectra with two absorption maxima in Band II of the spectra (Table 4), indicating three oxygen-substituents of the A-ring of the flavonol. The pseudomolecular masses of *m*/*z* 361 and 375 in the ESI+-spectra of the two compounds, respectively, is in agreement with the compounds eupatin (=3,3′,5-trihydroxy-4′,6,7-trimethoxyflavone) and casticin (=3′,5-dihydroxy-4′,3,6,7-tetramethoxyflavone) or chrysoplenetin (=4′,5-dihydroxy-3′,3,6,7-tetramethoxyflavone). Scopoletin, chrysoplenetin and casticin have previously been isolated from leaves of sweet wormwood together with chrysoplenetin and casticin [42]. 

Sweet wormwood, stalks: The chromatographic profile of the EtOH-extract of stalks was much like that of the leaves (Figure 4), with peaks **1**, **2**, **3**, **4** and **5** being present in both extracts (Table 4). Peak **6** revealed an absorbance spectrum in accordance with a caffeic acid moiety, and retention time higher than the dicaffeoyl quinic acid moieties, indicating a less hydrophilic compound. The presence of two fragment ions in the ESI+ spectrum at *m*/*z* 163 and 177 is in agreement with caffeic- and ferulic acid moieties, respectively, and the pseudomolecular ion at *m*/*z* 531 is in agreement with a caffeoyl-feruloylquinic acid. No flavonols were detected from the stalk extract.

Yarrow: The EtOH-extract of the yarrow flowers contained chlorogenic acid and dicaffeoylquinic acids, as detected in some of the above-described extracts as well. Peak **2** contained the same chromatographic and spectral characteristics as for luteolin 7-*O*-glucoside detected in roseroot. In analogy, peak 4 was annotated apigenin 7-*O*-glucoside. Peak **6** exhibited similar UV absorbance spectrum as peak **4**, and also revealed an ion in the mass-spectrum corresponding to apigenin. The difference between the molecular ion and the fragment of the aglycone, [M-271+H]^+^, was in agreement with malonylglucose, 248 amu, and thus the compound was assigned to be apigenin 7-*O*-malonylglucoside. The prolonged chromatographic retention compared to its non-acylated analogue is in accordance with this identification. Apigenin 7-malonylglucoside has rarely been reported from plants. It was previously reported from the moss species *Bryum capillare* (now *Ptychostomum capillare*) [43].

UHPLC-MS analysis was applied to characterize phenolic metabolites in the EtOH extracts. The consistence of the pseudomolecular ions from the two ESI-modes ([M+H]^+^/[M-H]^−^) were used to determine the specific molecular masses (*m*/*z*). In some cases, adducts ions of sodium ([M+Na]) was found to be more pronounced than the pseudomolecular ions. Adduct ions of potassium ([M+39]) was also used to confirm the molecular masses. Typical moiety-fragments of aglycones (e.g., *m*/*z* 303 for quercetin), sugars and aromatic acids (*m*/*z* 163 and 177 for caffeic and ferulic acid, respectively) were also detected in the positive mode. In negative MS-mode the base peak was often found as [2M-H]^−1^, which was in particular the case for compounds containing an aromatic acid moiety, e.g., chlorogenic acid and rosmarinic acid. Steckel (2019) describes this phenomenon to occur for compounds which are prone to complex formation and in cases of high concentration of the specific compound [44]. As the eluent contained formic acid some compounds appeared as adducts of formate, [M+HCOOH-H]^−1^, (M+45). This adduct formation seemed to depend on the sample type, and was found to be most pronounced in the cases of rhodionin and rhodiosin from roseroot. 

Antimicrobial activity: The summarized results of the disc diffusion tests are given in Table 5. Between the indicator organisms, the Gram-positive *S. aureus* is the more susceptible bacterium. The Gram-negative *E. coli* was not affected in our study. Plant metabolites, such as, e.g., flavonoids, are suggested to interact with the cell membrane of Gram-positive bacteria [45]. Considering the difference in structure and complexity from that of cell walls of Gram-negative, it is often suggested as an explanatory model for differences in susceptibility. However, results in our study suggest that the antimicrobial activity of the herb extracts tested is not directly related to bacterial membrane structure as the inhibitory action of extracts did not affect the Gram-negative indicator *E. coli*, but acted on the Atlantic salmon Gram-negative bacteria. Marine bacteria have evolved different membrane traits that adapt bacteria to marine ecosystems with anticipated membrane properties attributable to rapid changes during, e.g., growth conditions and growth stage [46,47]. The difference in growth condition among strains used in this study could be a factor that in part contributes to the above discrepancy between Gram-positive and Gram-negative bacteria. Considering that the activity of extracts (DCM, EtOH and H_2_O) was different for both *M. viscosa* and *A. wodanis* dependent on the growth media used (Table 5), it is worth exploring if the disk diffusion method is a reliable tool for the susceptibility testing of marine pathogens. Nevertheless, in our study, aqueous extracts exhibited less activity compared to organic solvents, which is in agreement with previous studies assessing bioactive compounds having antimicrobial activities [48]. The DCM- and EtOH-extracts showed greatest activity against the Gram-positive indicator organism and the Gram-negative Atlantic salmon pathogens among all tested bacteria (inhibition diameters ranged from 8→22 mm). The DCM-extracts of sweet wormwood stalk (harvested at September) and rosemary inhibited all strains except *E. coli*. The extract with the highest activity was different for each bacterium. DCM extract of hops gave high inhibition to *S. aureus,* whereas, hops (EtOH), maral root (EtOH), rosemary (DCM), sweet wormwood (EtOH, DCM) and yarrow (EtOH) inhibited to a lesser extent. For the Atlantic salmon pathogens, high antimicrobial activity against *T. finnmarkense* was shown from DCM extracts from hops flower cone and sweet wormwood leaves. Sweet wormwood stalk DCM extract inhibited *M. viscosa* the most. *A. wodanis* was affected by lower inhibition by several extracts (Table 5). Overall, the most promising herb extracts with antimicrobial activity against bacterial species in this study includes sweet wormwood (leaf and stalk from late harvest), rosemary leaves and hops cones.

Interestingly, among the two seasons tested for sweet wormwood, highest inhibition was obtained from extracts of late harvest season indicating that the different antimicrobial efficacies could relate to seasonal variations. The late season extracts (June vs. September) contained more phenolics. The difference in antimicrobial activity detected between crude extracts of different plant parts could be of further research interest in order to provide a scientific rationale for optimal utilization and efficacy of the herbs. Additional experiments aimed to evaluate the stability of selected extracts in aquatic environment and its effect on fish health must be evaluated. 

## 3. Materials and Methods

Dichloromethan (DCM) (Rathburn, HPLC-quality) was provided by Teknolab, Oslo, Norway. Technical 96% EtOH was provided from VWR, Oslo, Norway. Ferric chloride hexahydrate (Fluka), potassium hexacyanoferrate, 2,2-azino-bis (3-ethylbenzothiazoline-6-sulfonic acid) (ABTS), potassium peroxodisulfate, gum Arabic, sodium tungstate, phosphomolybdic acid, lithium sulphate, hydrochloric acid, 85% ortophosphoric acid, gallic acid, trolox, potassium phosphate, sodium chloride, sodium carbonate, bromine and gallic acid were provided from Merck, Oslo, Norway. In-house standards (PlantChem AS, Eiken, Norway) of kaempferol 3-glucoside and quercetin 3-glucoside were used for co-chromatography.

The herbs used in this work are listed in Table 6. Sweet wormwood was harvested twice during the growth season (in June and September). Plants were grown at NIBIO Apelsvoll (latitude 60.70024, longitude 10.86952) which is located within the Boreal, dry zone, USDA hardiness zone 6a, Köppen climate classification Dfc. Plant materials were dried at standard conditions (60 °C, 48–72 h), and milled on a FOSS mill CT 290 (Foss, Hilleroed, Denmark) down to particle size 1 mm. 

Dried and milled plant materials, 10–20 g DM, were mixed and extracted with 2 × 100 mL of DCM, EtOH and H_2_O, respectively, in a successive order giving in total six extraction steps for each sample. Extraction took place in 150 mL glass beakers with screw caps. Each extraction step was carried out with sonication at 40 kHz and at 35 °C for 30 min (Biltema, Lyngdal, Norway). Extracts were filtered between each extraction step (Cytiva Whatman, 2555 ½ folded filters 185 mm, VWR, Oslo, Norway), and filter cake was then re-extracted. The two extracts of the same solvent were combined and concentrated by use of a rotary evaporator (Büchi, Flawil, Switzerland). Concentrates were taken to dryness under a beam of N_2_-gass, and by use of lyophilisation (CoolSafe 4 ScanVac, ScanLaf AS, Alleroed, Denmark). In order to compare two extraction methods, some DCM-extractions of leaves and stalks of *Artemisia annua* were carried out by use of conventional Soxhlet extraction (Prat Dumas cartridges 33/38 × 130 mm, Teknolab, Oslo, Norway) with seven extraction cycles (total extraction time about 30 min). Extracts were concentrated and dried as previously described.

Total phenolic content was determined by use of the Folin–Ciocalteu (FC) method in accordance with the description of Waterman and Cole [49]. The FC-reagent was prepared as previously described [50]. Samples (100 μL) were mixed with 5 mL distilled H_2_O and 250 µL of the FC-reagent. After 1 min and before 8 min, 1 mL of a 20 g 100 mL^−1^ Na_2_CO_3_ solution was added, and time was recorded from zero. After 2 h, the absorbance was measured at 760 nm by use of an Agilent 8453 spectrophotometer (Agilent Technologies, Matriks, Oslo, Norway). Samples were processed against a standard curve of gallic acid (0–20 mg 100 mL^−1^) and results were given as gallic acid equivalents, mg GAE g^−1^. Samples that did not fit with the range of the standard curve were diluted with EtOH in the cases of DCM and EtOH-extracts, or with H_2_O in the case of H_2_O-extracts.

The TEAC assay (Trolox Equivalent Antiradical Capacity) was carried out following the procedures previously described by Re and co-workers [51]. 2,2-azino-bis (3-ethylbenzothiazoline-6-sulfonic acid) (ABTS) was dissolved in H_2_O to a 7 mM solution with potassium persulfate to a concentration of 2.45 mM. The solution was kept at ambient temperature for about 16 h. The ABTS^+^ solution was diluted with PBS (phosphate-buffered saline: 100 mM KH_2_PO_4_-buffer, pH 7.4 and 150 mM NaCl), to an absorbance of 0.70 (±0.02) at 734 nm. Samples were diluted so that, after the introduction of a 10 µL aliquot of each extract into the assay, they produced between 20–80% inhibition of the blank absorbance. After addition of 1.0 mL of diluted ABTS^+^ solution to 10 µL of extracts or trolox standards (final concentration 0–15 µM) in PBS, the absorbance reading was taken at 6 min. Appropriate PBS blanks were run in each assay. The percentage of inhibition of absorbance at 734 nm were expressed as µmol Trolox g^−1^ DM.

Ultra high-performance liquid chromatography (UHPLC) was performed on EtOH-extracts of all samples by use of an Agilent 1260 Infinity II instrument supplied with a diode-array detector (DAD) and a 6120 single-quadrupole mass detector with electrospray ionization (ESI) (Matriks AS, Oslo, Norway). Separation was achieved by use of a YMC-Triart C18-column, 100 × 2.0 mm, 1.9 µm (Teknolab, Oslo, Norway), with mobile phase consisting of 0.01% HCOOC (A) and acetonitrile (B). The gradient profile (%B in A) was: from 5 to 30% in 31 min, 30 to 100% in 9 min, 100 to 5% in 2 min. In the case of the hops-sample a modified gradient was used: from 15 to 33% in 7 min, 35 to 85% in 13 min, 85 to 100% in 10 min, and from 100 to 15% in 1 min. For both methods a post time of 1 min was included for reconditioning of the column. Mobile phase flow was set to 0.3 mL min^−1^, and injection volume to 5 µL. Electromagnetic spectra were collected in the range 230 to 600 nm, whereas chromatographic signals were obtained at 280, 320 and 360 nm with bandwidth 4 nm. ESI-MS was scanned in positive and negative mode in the range of 150 to 800 *m*/*z* with fragmentor at 40 V. Source settings were: gas temperature 300 °C, gas flow 10.4 L min^−1^, nebulizer 35 psi and capillary voltage 4 kV. Samples of the EtOH extracts were dissolved in 80% EtOH at concentrations of 1 mg mL^−1^, and filtered through syringe filters (Nylon, 0.45 µm) prior to analysis.

Stock solution (*w/v*) of each herb extract was prepared by dissolving 10 mg of the dried sample in 1000 µL of respective solvents. H_2_O-soluble extracts were re-dissolved in nuclease free H_2_O (Ambion^®^, ThermoFisher Scientific, Oslo, Norway), DCM extracts were re-dissolved in DCM (VWR International AS), and alcohol extracts were re-dissolved in 80% EtOH (VWR International AS, Oslo, Norway) by vortexing. Any un-dissolved pellets were sonicated for 1 min and vortexed thoroughly until dissolved. 

The antimicrobial activity was tested against two indicator organisms, the Gram-positive bacteria *S. aureus* ATCC29213 and the Gram-negative bacteria *E. coli* ATCC25922, in addition to the three Gram-negative Atlantic salmon pathogens *M. viscosa* 06/09/139, *T. finnmarkense* NCIMB 15238 and *A. wodanis* 06/09/139. Antimicrobial activity of the herb extracts was performed using the disk diffusion protocol, based on the EUCAST guidelines for antimicrobial susceptibility testing (version 9.0, January 2021). Indicator organisms were pre-cultured in Mueller Hinton (MH) broth (Oxoid, lot no: 3359347) overnight in a rotary shaker at 37 °C and concentration adjusted by using the 0.5 McFarland standard. The Atlantic salmon pathogens were pre-cultured in marine broth (Difco™ ThermoFisher Scientific, Oslo, Norway) and Luria-Bertani broth (Sigma-Aldrich, St. Louis, MO, USA) in a rotary shaker at 12 °C for 2 days. After which, a spectrophotometer (A600 nm) was used to measure optical density (OD) and cultures at OD 0.8 was chosen for the experiment. Cultures were diluted 10^−5^ and 100 µL suspension was plated into Marine agar and Luria-Bertani agar, except *T. finnmarkense* which grows only in marine broth. Antimicrobial activity was tested applying 20 µL (200 µg) of the working solution to each filter disc (6 mm diameter, Whatman AA disc, CAT no.2017-006). Disks were allowed to dry for 20 min and placed on the previously inoculated agar plates with selected bacteria. Six discs per plate were arranged and solvents without extracts acted as negative controls. Plates were incubated for 20 hrs at 37 °C for indicator organisms and 3 days at 12 °C for Atlantic salmon bacteria. Antimicrobial activity was determined by measuring the diameter of the inhibition zone around the disc in millimeters.

**Statistics**. Calculation of means and standard deviations (STDA) were performed in Microsoft Excel software (2016). Two-sample t-tests were performed by use of Med Calc’s calculator (https://www.medcalc.org/calc/comparison_of_means.php accessed 5 August 2022). Combined standard deviations were calculated by use of online statistics (http://www.obg.cuhk.edu.hk/ResearchSupport/StatTools/CombineMeansSDs_Pgm.php accessed on 5 August 2022).

## 4. Conclusions

Extraction of herbs with DCM gave low yields (<4%) except for hops, rosemary and sweet wormwood leaves. Hydrophilic extracts (EtOH and H_2_O) revealed higher extraction yields and the highest content of phenolics and radical scavenger capacities. This preliminary study demonstrates promising antimicrobial activity of several herbs against bacterial Atlantic salmon pathogens. DCM, and to a less extend EtOH, extracts possessed the highest antimicrobial activities. However, the discrepancy between the inhibition of a Gram-positive indicator organism and Gram-negative Atlantic salmon bacteria suggests that better standardization and reference tools need to be developed for susceptibility tests of marine bacteria. Future investigations are designed to understand the bactericidal concentrations of single or combination of potential extracts on salmon pathogens. In order to meet with the requirement of a phytogenic feed, data from our study suggest that enhanced antioxidative and antimicrobial properties could be also obtained through a combination of a DCM extract with high antimicrobial activity with a EtOH or H_2_O extract with high antioxidative capacity.

## Figures and Tables

**Table 1:** The percentage extraction yields (mean ± SD) on a dry weight basis of nine herb. All extracts were made by ultrasound-assisted extraction (UAE). Extraction was performed in a sequential manner (DCM followed by EtOH and finally by H_2_O).

		Yields (%)			
	*n*	DCM	EtOH	H_2_O	Total
Hops (*H. lupulus*)	12	11.6 ± 3.9	5.1 ± 2.1	12.3 ± 3.8	28.9 ± 4.6
Maral root, leaves (*L. carthamoides*)	5	2.9 ± 0.5	3.4 ± 1.0	19.0 ± 1.2	25.3 ± 0.9
Maral root, root (*L. carthamoides*)	5	1.1 ± 0.9	2.7 ± 1.7	12.1 ± 3.5	16.0 ± 2.3
Mint (*M. piperita*)	5	3.3 ± 0.2	8.5 ± 1.6	20.2 ± 2.9	32.0 ± 7.5
Oregano (*O. vulgare*)	30	2.7 ± 0.6	5.6 ± 4.1	26.1 ± 7.0	34.3 ± 11.5
Purslane, (*P. oleracea* ssp. sativa)	8	3.1 ± 0.7	9.2 ± 2.0	19.0 ± 5.1	31.3 ± 7.4
Rosemary (*R. officinalis*)	7	8.8 ± 3.4	4.9 ± 1.7	10.9 ± 5.6	24.5 ± 4.5
Roseroot (*R. rosea*)	7	1.3 ± 0.6	2.2 ± 5.5	5.8 ± 2.4	9.3 ± 3.9
Sweet wormwood, leaves (*A. annua*)	16	6.1 ± 3.3	2.8 ± 1.4	9.1 ± 6.5	18.0 ± 4.9
Sweet wormwood, stalks (*A. annua*)	16	1.2 ± 4.2	1.8 ± 1.3	5.9 ± 5.7	8.9 ± 4.6
Yarrow (*A. millefolium*)	10	3.8 ± 0.7	3.2 ± 1.3	12.0 ± 2.3	19.0 ± 4.4

*n*: number of lines/varieties.

**Table 2:** Scavenging capacities of the ABTS-radical measured as Trolox equivalents (TEAC) and total phenolic content of dried extracts from herbs successively extracted with DCM followed by EtOH and H_2_O.

		Radical Scavenging Capacities			Total Phenolics	
		(µmol Trolox g^−1^ DM Extract)			(mg GAE g^−1^ DM Extract)	
	*n*	DCM	EtOH	H_2_O	EtOH	H_2_O
Hops	4	922 ± 130	2045 ± 175	1157 ± 437	277 ± 49	122 ± 43
Maral root, leaves	1	161 ± 8	1070 ± 235	654 ± 46	89 ± 24	92 ± 4
Maral root, root	1	168 ± 41	673 ± 272	557 ± 35	61 ± 16	64 ± 1
Mint	5	306 ± 78	607 ± 299	1460 ± 462	90 ± 13	181 ± 52
Oregano	24	256 ± 79	1033 ± 420	2723 ± 399	238 ± 71	326 ± 75
Purslane	8	103 ± 56	487 ± 144	120 ± 28	31 ± 9	8 ± 4
Rosemary	7	945 ± 238	926 ± 292	812 ± 178	206 ± 114	124 ± 19
Roseroot	7	454 ± 90	1161 ± 318	1334 ± 468	256 ± 34	143 ± 45
*S. wormwood*, leaves, June	6	142 ± 32	399 ± 73	151 ± 42	46 ± 15	22 ± 7
*S. wormwood*, leaves, September	6	195 ± 51	1057 ± 230	307 ± 111	194 ± 64	61 ± 12
*S. wormwood*, stalks, June	6	206 ± 98	265 ± 49	66 ± 12	32 ± 15	11 ± 3
*S. wormwood*, stalks, September	6	571 ± 173	532 ± 215	89 ± 15	100 ± 26	22 ± 6
Yarrow	10	59 ± 27	896 ± 346	615 ± 107	117 ± 49	105 ± 19

*n*: numbers of line/varieties.

**Table 3:** Combination of extraction yields and radical scavenging capacities.

	µmol TEAC g^−1^ DM Plant		
	DCM	EtOH	H_2_O
Hops	107	104	142
Maral root, leaves	5	37	124
Maral root, root	2	18	68
Mint	10	52	294
Oregano	7	57	710
Purslane	3	45	23
Rosemary	83	45	88
Roseroot	6	26	78
*S. wormwood*, leaves	12	29	28
*S. wormwood*, stalks	7	9	5
Yarrow	2	28	74

**Table 4:** Chromatographic and spectral characteristics of major peaks in the UHPLC-DAD-ESIMS chromatograms of the EtOH extracts of the herbs. Peak numbering is restarted for each herb sample.

Species	*#*	*t*_R_ (min)	λ_max_ (nm)	ESI-pos (*m*/*z*)	ESI-neg (*m*/*z*)	Putative Identification
Hops	1	1.54	286, 324	163, 355	353	chlorogenic acid
*Humulus lupulus*	2	5.66	256, 265sh, 354	465	463	quercetin 3-*O*-glucoside
	3	6.49	269, 348	449	447	kaempferol 3-*O*-glucoside
	4	14.60	240, 370	371	369	xanthohumol B
	5	15.36	248, 290, 366	341	339	desmethylxanthohumol
	6	16.97	244, 290sh, 370	299, 355	353	xanthohumol C
	7	19.28	246, 284, 322, 360sh	349	347	cohumulone
	8	20.10	242, 286, 322, 360sh	363	361	humulone
	9	20.25	242, 290, 322, 360sh	363	361	adhumulone
	10	22.48	244, 276, 334	401	399	colupulone
	11	23.18	244, 276, 334	415	413	lupulone / adlupolone
Maral root, leaves	1	9.58	234, 300sh, 326	163, 355	353	chlorogenic acid
*Leuzea carthamoides*	2	16.47	238sh, 260, 270sh, 358	319, 481	479	6-hydroxyquercetin-*O*-hexoside
	3	19.63	236, 274, 346, 370sh	303, 465	463	6-hydroxykaempferol-*O*-hexoside
	4	20.07	258, 364	303, 495	493	methoxyquercetin-hexoside
	5	20.55	235sh, 258, 352	333, 495	493	6-methoxyquercetin-*O*-hexoside
	6	23.14	238sh, 264, 346	317, 479	477	6-methoxykaempferol-*O*-hexoside
Maral root, root	1	9.55	234, 300sh, 326	163, 355	353	chlorogenic acid
*Leuzea carthamoides*	2	18.52	250, 352	317, 479	477	isorhamnetin-*O*-hexoside
	3	21.14	234, 300sh, 326	163, 517	515	di-caffeoyl quinic acid isomer
	4	22.09	236, 300sh, 328	163		di-caffeoyl quinic acid isomer
	5	23.75	236, 300sh, 326	163, 517	515	di-caffeoyl quinic acid isomer
	6	24.24	236, 300sh, 326	163	631	caffeic acid derivative
	7	28.60	236, 300sh, 326	163	793	caffeic acid derivative
Mint	1	17.52	236, 282, 328sh	289, 451, 597	595	eriodictyol 7-*O*-rutinoside
*Mentha piperita*	2	19.57	254, 266, 348	287, 449, 595	593	luteolin 7-*O*-rutinoside
	3	20.15	254, 266, 346	287, 463	461	luteolin 7-*O*-glucuronide
	4	22.13	270sh, 284, 332	303, 465, 611	609	quercetin 3-*O*-rutinoside
	5	23.20	236, 295sh, 330	163, (361)	359; 719	rosmarinic acid
Oregano	1	18.05	264, 300sh	(123)	153, 437, 875	oreganol A
*Origanum vulgare*	2	21.72	258, 270sh	(123)	421, 843	4′-*O*-β-d-glucopyranosyl-4′-hydroxybenzyl protocatechuate
	3	22.68	262, 300sh	(123)	421, 451, 903	oreganol B
	4	23.25	290sh, 330	(163, 361)	359, 719	rosmarinic acid
Purslane	1	15.90	290sh, 342	163, 197, 520	401, 518	oleracein W
*Portulaca oleracea*	2	18.57	300sh, 332	163, 183, 342, 504	502	oleracein A
	3	23.15	266, 286, 296, 320sh	183, 693	451, 691	unknown
	4	28.40	302sh, 334	227, 710	310, 708	oleracein-derivative (FGIF)
	5	33.08	300sh, 334	195, 275, 293, 351	327	caffeic acid derivative
Rosemary	1	10.36	240, 295sh, 322	181	179	caffeic acid
*Rosmarinus officinalis*	2	20.11	254, 266, 338	183, 449	447	luteolin-7-*O*-glucoside
	3	21.22	252, 272, 334	163, 183, 317, 479	477	nepitrin
	4	22.08	244, 298sh, 328	163, 355, 499	353, 515	dicaffeoylquinic acid
	5	23.29	238, 295sh, 328	163, 361	359, 719	rosmarinic acid
	6	30.10	248, 268, 340	317	315	nepetin
	7	34.40	252, 300sh, 336	163, 301	299, 313, 627	unknown
Roseroot	1	16.61	236, 264	446	473	rosavin (=cinnamyl-(6′-ara)-glc)
*Rhodiola rosea*	2	26.23	236, 276, 330, 382	303, 449, 611	609, 645	rhodiosin (=herbacetin 7-glc-rha)
	3	26.94	234, 276, 330, 382	303, 449	301, 447	rhodionin (=herbacetin 7-rha)
Sweet wormwood, leaves	1	9.31	294, 330	163, 244, 355	353, 389, 399	chlorogenic acid
*Artemisia annua*	2	14.43	240, 300sh, 324	177, 369	367, 735	feruloylquinic acid
	3	15.56	232, 296, 344	193, 215	191	scopoletin
	4	19.28	232, 276, 322	165	163	unknown
	5	22.15	242, 300sh, 328	163, 272, 499, 517	515	dicaffeoylquinic acid
	6	23.93	244, 300sh, 328	163, 272, 499, 517	515	dicaffeoylquinic acid
	7	35.12	258, 270sh, 350	361	359	eupatin
	8	36.70	256, 270sh, 348	375	373	casticin/chrysoplenetin
Sweet wormwood, stalks	1	9.74	294, 330	193, 355	191, 353, 399	chlorogenic acid
*Artemisia annua*	2	14.57	240, 300sh, 324	177, 369	367, 735	feruloylquinic acid
	3	15.70	234, 296sh, 344	193, 215	191	scopoletin
	4	22.17	244, 298sh, 328	163, 272, 499, 517	515	dicaffeoylquinic acid
	5	23.91	244, 300sh, 328	163, 272, 517	159, 515	dicaffeoylquinic acid
	6	26.38	242, 300sh, 326	163, 177, 513, 531	529	caffeoyl-feruloylquinic acid
Yarrow, flowers	1	9.55	240, 300sh, 326	163, 355, 551	353, 707	chlorogenic acid
*Achillea millefolium*	2	20.07	254, 266sh, 348	287, 449	447	luteolin-7-*O*-glucoside
	3	22.08	242, 300sh, 326	163, 517	515	dicaffeoylquinic acid
	4	22.96	236, 266, 334	271, 433	431	apigenin-7-*O*-glucoside
	5	23.87	244, 300sh, 328	163, 517	515	dicaffeoylquinic acid
	6	27.34	236, 266, 336	271, 519	269, 517	apigenin 7-*O*-malonylglucoside
	7	29.68	254, 266, 348	287	285	luteolin
	8	33.99	236, 266, 336	271	269	apigenin

sh = shoulder; () = weak signal.

**Table 5:** Disk diffusion antimicrobial activity of herb extracts made in sequential order; DCM followed by EtOH and finally H_2_O. Bacterial species were grown on Mueller Hinton agar (MH), Marine agar (MA) and Luria Bertani agar (LB). Bacteria and growth medium used: 1. *Staphylococcus aureus* on MH, 2. *Tenacibaculum finnmarkense* on MA, 3. *Moritella viscosa on MA*, 4. *Moritella viscosa* on LB, 5. *Aliivibrio wodanis* on MA. 6. *Aliivibrio wodanis* on LB. Inhibition zone values are represented as: mean (*n*) ± SD, where n is the number of lines/varieties tested. Susceptibility is considered >7 mm (disk is 6 mm). Extracts with no activities are left out. *Escherichia coli* was not susceptible to any extracts and is not included in the table.

	Extract	1	2	3	4	5	6
Hops, cones	DCM	18 ± 4.7, *n* = 4	22.3 ± 5.5, *n* = 4	-	8.5 ± 1.0, *n* = 4	8.5, *n* = 2	8.7 ± 0.6, *n* = 3
	EtOH	8.5 ± 1.0, *n* = 4	-	-	-	-	10, *n* = 4
Maral root, leaves	DCM	-	11, *n* = 1	-	-	-	-
	EtOH	14, *n* = 1	-	8, *n* = 1	-	-	-
Maral root, root	DCM	-	9.5, *n* = 2	-	-	-	-
	H_2_O	-	-	-	-	-	8.5, *n* = 1
Oregano, leaves	EtOH	-	8.8 ± 0.45, *n* = 5	-	-	-	-
	H_2_O	-	-	-	-	-	9.2 ± 1.0, *n* = 13
Rosemary, leaves	DCM	11.9 ± 0.8, *n* = 7	14.9 ± 1.4, *n* = 7	13.9 ± 1.1, *n* = 7	10.4 ± 4.4, *n* = 7	11 ± 2.0, *n* = 6	13.8 ± 2.5, *n* = 6
	EtOH	-	10.8 ± 1.1, *n* = 5	-	8, *n* = 4	-	13 ± 1.6, *n* = 5
Roseroot, roots	H_2_O	-	-	8, *n* = 5	9.3 ± 1.2, *n* = 3	-	9.5, *n* = 2
Sweet wormwood, leaves June	DCM	-	12.2 ± 2.8, *n* = 5	-	-	-	-
	EtOH	9.3 ± 0.5, *n* = 4	-	-	-	-	-
Sweet wormwood, stalks June	DCM	-	-	17.5 ± 2.6,*n* = 4	19.5 ± 1.7,*n* = 4	-	-
Sweet wormwood, leaves September	DCM	-	19.3 ± 1.0,*n* = 6	-	-	-	-
Sweet wormwood, stalks September	DCM	12.2 ± 1.0, *n* = 6	12 ± 2.0, *n* = 3	25, *n* = 6	25, *n* = 6	8, *n* = 5	9 ± 0.6, *n* = 6
	EtOH	-	-	-	20, *n* = 6	-	-
Yarrow, flowers	DCM	-	12.6 ± 3.9, *n* = 7	-	-	-	-
	EtOH	11.7 ± 1.0, *n* = 10	-	-	-	-	-

**Table 6:** Overview of plant species and plant parts used for solvent extraction.

Species	Plant Family	Plant Parts Used in Experiment
Oregano (*Origanum vulgare*)	Lamiaceae	Leaves, harvested prior to flowering
Yarrow (*Achillea Millefolium*)	Asteraceae	Flowers
Peppermint (*Mentha pipperita*)	Lamiaceae	Leaves, harvested prior to flowering
Hops (*Humulus lupulus*)	Cannabaceae	Cones
Rooseroot (*Rhodiola rosea*)	Crassulaceae	Rhizome
Maral root (*Leuzea carthamoides*)	Asteraceae	Leaves and root
Sweet wormwood (*Artemisia annua*)	Asteraceae	Leaves (harvested prior to flowering), stalks
Purslane (*Portulaca oleracea*)	Portulacaceae	Leaves, harvested prior to flowering
Rosemary (*Rosmarin officinales*)	Lamiaceae	Leaves, harvested prior to flowering

## Data Availability

Not applicable.

## Sample Availability

Samples of the compounds kaempferol 3-glucoside and quercetin 3-glucosid are available from the authors.
